# Carvacrol Arrests the Proliferation of Hypopharyngeal Carcinoma Cells by Suppressing Ornithine Decarboxylase and Hyaluronidase Activities

**DOI:** 10.3389/fnut.2022.857256

**Published:** 2022-04-08

**Authors:** Kaneez Fatima, Suaib Luqman, Abha Meena

**Affiliations:** ^1^Bioprospection and Product Development Division, CSIR-Central Institute of Medicinal and Aromatic Plants, Lucknow, India; ^2^Academy of Scientific and Innovative Research (AcSIR), Ghaziabad, India

**Keywords:** carvacrol, ornithine decarboxylase, hyaluronidase, FaDu, biomarkers, molecular targets

## Abstract

Carvacrol, a monoterpene known for its pharmacological activities, is present in the essential oil of *Origanum majorana, Origanum vulgare, Thymus vulgaris*, and *Lippia graveolens*. It is used in food as a flavoring and preservative agent in cosmetics and medicines because of its useful bioactivities in clinical practice. However, carvacrol was not much explored for its anticancer potential. Targeting enzymes involved in carcinogenesis, such as ornithine decarboxylase (ODC), cyclooxygenase-2 (COX-2), lipoxygenase-5 (LOX-5), and hyaluronidase (HYAL) by monoterpenes are amongst the efficient approaches for cancer prevention and treatment. In this study, the efficacy of carvacrol was investigated against deregulated cancer biomarkers/targets in organ-specific human cancer cell lines (FaDu, K562, and A549) utilizing *in vitro, in silico*, and *in vivo* approaches. The efficacy of carvacrol was evaluated on human cancer cell lines using neutral red uptake (NRU), sulpho rhodamine B (SRB), and 3-(4,5-dimethylthiazol-2-yl)-2,5-diphenyl tetrazolium bromide (MTT) assays. The mechanistic study was carried out in cell-based test systems. Further, the potency of carvacrol was confirmed by the quantitative real-time PCR analysis and molecular docking studies. The *in vivo* anti-tumor potential of carvacrol was performed on mice S-180 model, and the toxicity examination was accomplished through *in silico* approach. Carvacrol significantly impeded the growth of FaDu, K562, and A549 cell lines with IC_50_ values ranging from 9.61 ± 0.05 to 81.32 ± 11.83 μM. Further, the efficacy of carvacrol was explored against different cancer targets in FaDu, K562, and A549 cell lines. Carvacrol inhibits the ODC, COX-2, LOX-5, and HYAL activities in FaDu cell line and ODC, COX-2, and HYAL activities in K562 cell line. The results were validated by expression analysis revealing the downregulation of the targeted gene with a significant change in the transcript level of ODC and HYAL in FaDu cell line with a fold change of 1.56 and 1.61, respectively. A non-significant effect of carvacrol was observed on the downstream signaling pathway of PI3K and HIF-1α/vascular endothelial growth factor (VEGF) in FaDu cells. The cell cycle, reactive oxygen species (ROS), mitochondrial membrane potential (MMP), and Annexin V-fluorescein isothiocyanate (FITC) experiments demonstrate that carvacrol induces apoptosis of FaDu cells. Further, the potency of carvacrol was also evaluated *in vivo* on mice S-180 tumor model, wherein it inhibits tumor growth (72%) at 75 mg/kg body weight (bw). ADMET studies predicted carvacrol as a safe molecule. Overall, carvacrol delayed the growth of FaDu, K562, and A549 cell lines by targeting enzymes involved in the carcinogenesis process. The existence of one hydroxyl group at the para position of carvacrol could be responsible for the anti-proliferative activity. Thus, carvacrol could be used as a pharmacophore to develop a safe and effective multi-targeted anti-cancer medicament.

## Introduction

Carvacrol (5-isopropyl-2-methyl phenol) is a natural cyclic phenolic monoterpene in aromatic plants. It is abundantly available in the essential oils of ajwain, marjoram, oregano, wild bergamot, pepperwort oil, and thyme oil (1, 2). Food and Drug Administration (FDA) recognized carvacrol as a safe flavoring additive in baked foods, sweets, chewing gum, and beverages (3). In addition, it is used in agriculture to control pests (4). Carvacrol and thymol treat beehives against the varroa mite without harming the bees (5). Besides, carvacrol exhibits an array of pharmacological properties, such as anti-inflammatory, anti-microbial, anti-mutagenic, anti-platelet, anti-oxidant, analgesic, anti-elastase, anti-angiogenic, anti-tumor, cell-protective, insecticidal, anti-parasitic, and AChE inhibitor activities (6, 7). It is a volatile molecule, and its vapor phase possesses anti-microbial activity. The effectiveness of carvacrol against the resistant species has been revealed with 0.015–0.03% v/v minimum inhibitory concentration (MIC) values for carvacrol and 0.06–0.125% MIC values for essential oils having a good percentage of carvacrol (8, 9). Liolios et al. stated that carvacrol and *Origanum dictamus* L. essential oil with phosphatidyl choline-based liposomes showed improved anti-oxidant and anti-microbial potential (10). The essential oils contain an excellent quantity of carvacrol that exhibit strong anti-oxidant properties comparable with vitamin E, butyl hydroxyl toluene (BHT), and ascorbic acid (11). Furthermore, carvacrol induces a significant hepatoprotective action and improves the activity of enzymatic anti-oxidants (glutathione peroxidase, superoxide dismutase, and catalase), and also non-enzymatic anti-oxidants levels [reduced glutathione, vitamin C, and vitamin E; (12)].

The anti-proliferative potential of carvacrol has been described in different cancer cell lines, such as non-small lung cancer (A549), chronic myeloid leukemia (K562), human metastatic breast cancer (MDA-MB 231), murine B16 melanoma, human cervical (HeLa, SiHa), and prostate cancer (PC-3). Carvacrol induces growth inhibition in HeLa and SiHa cell lines and significantly diminishes DNA damage in K562 cells (13–19). It activates PARP and suppresses cyclooxygenase-2 (COX-2) inflammation, thereby reducing the amount of estrogen in rats without directly binding to estrogen receptors (ERs) (20, 21). It exhibits chemotherapeutic effect in DMH (1,2-dimethylhydrazine) persuaded colon carcinogenesis, shown by its role in reducing pre-neoplastic lesions, detoxifying the carcinogen, anti-proliferative, and anti-inflammatory activities. Besides, it weakens the cancer cells upon X-radiation without affecting normal cells; thus, it is called a radio-modulator (22). Owing to its high significance, researchers have synthesized carvacrol analogs and reported many derivatives of carvacrol and thymol containing 5-phenyl-2-furan motifs (23, 24). These derivatives exhibit auspicious anti-tumor activity against drug-resistant human hepatocellular carcinoma (Bel-7402) and drug-sensitive KB cells. Surprisingly, carvacrol derivatives exhibit better biological activity than thymol derivatives (25). The structure-activity relationship of carvacrol derivatives studied through the e-pharmacophore modeling approach recognized carvacrol as a prospective lead for the mycobacterial enzyme (26).

The anti-proliferative activity of carvacrol has been reported on various human cancer cell lines; however, its anti-cancer efficacy on targets involved in different cancer hallmarks and on FaDu cell line has not been reported so far. Therefore, this study was carried out to explore the efficacy of carvacrol on the selected targets of proliferation [dihydrofolat-ereductase (DHFR), ornithine decarboxylase (ODC), histone deacetylase-6 (HDAC-6), phosphotidyl inositol-3 kinase (PI3K), protein kinase B (AKT), and mammalian target of rapamycin (mTOR)], inflammation [lipoxygenase-5 (LOX-5) and cyclooxygenase-2 (COX-2)], angiogenesis [vascular endothelial growth factor (VEGF), hypoxia-inducible factor 1-alpha (HIF-1α), and hyaluronidase (HYAL)], metastasis and invasion [cathepsin D (CATD)], and anti-proliferative activity on various organ-specific human cancer cell lines, such as FaDu, A549, MCF-7, COLO-205, K562, WRL-68, and HEK-293. Further, the effect was explained by the cell cycle, real-time expression analysis, reactive oxygen species (ROS), mitochondrial membrane potential (MMP), and Annexin V-FITC assays. Besides, the effectiveness of carvacrol was also tested on mice S-180 tumor model and the drug-likeness properties were examined using *in silico* prediction tools.

## Materials and Methods

### Chemicals

Dimethyl sulfoxide (DMSO, D8418), formaldehyde (61783750001730), and isopropanol (1.94524.2521) were purchased from Merck Ltd., Mumbai, India. 3-(4,5-dimethylthiazol-2-yl)-2,5-diphenyl tetrazolium bromide (MTT, M2128), neutral red uptake (NRU, N4638), sulpho rhodamine B (SRB, 230162), antibiotic-antimycotic (Ab/Am, A5955) solution, HEPES (H4034), Propidium iodide (PI, P4170), RNase A (R5000), DCF-DA (D6883), phosphate-buffered saline (PBS, P3813), Rhodamine 123 (R8004), trypsin (93615), ODC (O3001), CATD (C8696), hemoglobin (H2625), sodium bicarbonate (S6297), dihydrofolate reductase (D6566), HYAL (H3884), hyaluronic acid (HA, 924474), cyclooxygenase-2 (COX-2, C0858), methotrexate (MTX, 06563), pepstatin A (P4265), α-difluoro methyl ornithine (DFMO, D193), N-acetyl cysteine (NAC, A7250), celecoxib (32097), zileuton (Z4277), and carvacrol (282197) were purchased from Sigma-Aldrich, Bengaluru, India. Dulbecco's minimal essential media (DMEM) (12100-061), and fetal bovine serum (FBS, 10082147) were procured from Gibco, Thermo Fisher Scientific India Pvt. Ltd., Mumbai, India. LOX-5 (60402) was purchased from Cayman Chemical Company, USA. Sodium/potassium phosphate (RM 257, RM 249) and trichloroacetic acid (RM 6274) were procured from Himedia Ltd, Mumbai, India.

### Anti-Proliferative Activity of Carvacrol

The anti-proliferative activity of carvacrol (5-isopropyl-2-methyl phenol, 98%) on different human cancer cell lines was performed by SRB, NRU, and MTT assays. The cell lines used for the present study were K562 (erythroid leukemia), COLO-205 (colon carcinoma), FaDu (hypopharyngeal carcinoma), MCF-7 (breast ER+ve adenocarcinoma), WRL-68 (hepatocellular carcinoma), A549 (lung carcinoma), and HEK-293 (embryonic kidney). These cell lines were purchased from the National Center for Cell Sciences (NCCS), Pune, India, except FaDu, which Dr. Sarkar endowed from CSIR-Central Drug Research Institute, Lucknow, Uttar Pradesh, India.

The SRB assay was carried out by following the method reported by Skehan et al. (27) with a slight modification (28). In 96 well plates, approximately ~2 ×10^3^ cells per well were seeded. Then, cells were treated with different concentrations (100 nM−100 μM) of carvacrol and incubated for 72 h. Approximately 20% of TCA was added in treated plates to precipitate cellular proteins, followed by washing cells with tap water and adding 10 μl SRB dye (0.4%) in each well of the plate. Then, the plate was washed with 1% acetic acid to remove the unbound dye. The bound dye was dissolved in 200 μl tris base (10 mM). The absorbance was finally measured at 515 nm using a spectrophotometer (Multiskan^TM^ Go SkanIt Software, version 4.0, Thermo Fisher Scientific, Waltham, MA, USA).

The NRU assay was carried out by Babich and Borenfreund (29) method. Similar to the SRB assay, 5 μl NRU dye was added to the treated plate. Then, cells were washed with fixative solution after 3 h of incubation; further, the bound dye was solubilized in a dissolving solution. The absorbance was measured at 540 nm (29).

The MTT assay was performed following the protocol as Mosmann (30) determined. In 96 well plates, approximately 2 × 10^3^ cells/well were seeded and treated with different carvacrol concentrations (100 nM−100 μM) after 24 h. The next day, 10 μl (500 μg/ml) of MTT dye was added to each well of the plate and further incubated for 4 h. After that, the formazan crystal was dissolved in 100 μl DMSO and absorbance was measured at 570 nm. The SRB, NRU, and MTT assays were calculated using percentage cytotoxicity concerning control (30).

#### Dihydrofolate Reductase and ODC Assays

Selected cell lines were treated with the different concentrations of carvacrol, and cell lysate was prepared with radioimmuno precipitation assay (RIPA) buffer. Protein was estimated by nanodrop before being used for the enzyme assays.

In one set, ~10 μg of cell lysate was incubated with a reaction cocktail (2.3 mM DHFA, 0.11 mM β-NADPH in 50 mM potassium phosphate buffer, and pH 6.5), while another set was incubated in the reaction cocktail containing 0.1% bovine serum albumin (BSA) solution. The reaction mixture was mixed, and the kinetic study was performed at 340 nm for 5 min. The results were calculated using percentage inhibition concerning control (31). The ODC activity was determined by the modified method reported by Luqman et al. (32). Approximately 10 μg of cell lysate was added to the reaction cocktail (2.5 mM β-mercaptoethanol, 1.5 mM EDTA, 75 nM pyridoxal-phosphate, 3 mM L-ornithine hydrochloride in 150 mM potassium phosphate buffer, and pH 7.1). Approximately, 400 μl of 1 M perchloric acid was used to terminate the reaction, and NaOH (4N) was added to the supernatant. Further, 1-pentanol was added after centrifugation. Then, the upper organic phase was transferred to a microcentrifuge tube with 200 μl sodium borate, 200 μl TNBS, and 400 μl DMSO. Further, it was centrifuged, and 60 μl of supernatant was taken in plate, and absorbance was measured at 412 nm. The graph was plotted in the terms of percentage inhibition.

#### COX-2 and LOX-5 Assays

Cyclooxygenase-2 (COX-2) activity was measured as reported by Kulmaczand Lands (33). The reaction cocktail consists of Tris-HCl buffer (100 mM and pH 8.0), EDTA (3 μM), hematin (15 μM), and cell lysate. After incubation, arachidonic acid (AA) and N,N,N′,N′-tetra methyl-p-phenylenediamine (TMPD) was added to 96 well plates to initiate the reaction. The reaction mixture was immediately auto mixed using the Multiscan Go of Thermo Scientific spectrophotometer, and absorbance was measured at 590 nm (33). The enzyme inhibition was calculated concerning control. For measuring the LOX-5 activity, different concentration of carvacrol/inhibitor (zileuton) was incubated with cell lysates. Approximately 40 μM of AA was mixed to initiate the reaction, and the plate was kept for 4 min at room temperature. The reaction was terminated by adding an equal ratio of reagent 1 (4.5 mM FeSo4 in HCl) and reagent 2 (3% NH_4_SCN). The absorbance was taken at 480 nm using a spectrophotometer (34). The enzyme inhibition was compared concerning control.

#### HYAL and CATD Activity Assays

The HYAL assay was performed by the standard reported protocol (35). Approximately 10 μg of cell lysate added in enzyme diluent buffer (77 mM sodium chloride, 20 mM sodium phosphate, 0.01% albumin, and pH 7.0 at 37°C). The reaction mixture was incubated for 45 min after adding HA (substrate). The mixture was distributed in three parts, each well of the plate containing 25 μl followed by the addition of 125 μl of diluent buffer (79 mM acetic acid, 24 mM sodium acetate, 0.1% albumin bovine, and pH 3.75). The absorbance was measured at 600 nm after 10 min incubation. The graph was shown in the terms of percentage inhibition. Hemoglobin was used as a substrate to determine CATD activity (36). Cell lysate (10 μg) was incubated with a reaction cocktail (2.5% of hemoglobin, deionised water, 400 mM citrate buffer, and pH 2.8) for 30 min at 37°C; the reaction was terminated by adding 5% TCA. Then, 200 μl of supernatant was taken in plate after centrifuge and absorbance were measured at 280 nm.

### Molecular Docking and Real-Time Expression Analysis

Auto-Dock Vina 1.1.2 software (Molecular Graphics Lab at The Scripps Research Institute, La Jolla, USA) was used to observe the molecular interaction of the ligand with the receptor (protein/enzyme) (37). The interacting amino acids and hydrophobic interactions were visualized by using the Discovery studio v3.5 visualiser [Accelrys Inc., USA, 2013; (38)]. The respective cells were treated with carvacrol/inhibitor, and after 24 h, RNA was isolated from the cells using RNAiso Plus reagent (Takara Bio Inc., Shiga, Japan). From 1–3 μg of RNA, cDNA was synthesized using the cDNA Reverse Transcription Kit (Applied Biosystems^TM^, USA). Primer Express^®^ Software v3.0 was used to design the forward and reverse primer of the targeted gene. Then, the primers were synthesized from Integrated DNA Technologies, Inc., USA (Table 1). After cDNA synthesis, 2 μl of template cDNA, 2.5 μl (10X) buffer, 0.5 μl DNA polymerase, 1 μl (10 mM) dNTPs, 1 μl forward primer, 1 μl reverse primer, 2 μl deionised water added to each well in 384-well plate. Glyceraldehyde 3-phosphate dehydrogenase (GAPDH) was used as the internal control. The real-time assay was accomplished on a real-time PCR machine, and the relative gene expression was calculated concerning non-treated samples (39).

**Table 1:** Primer sequence and size used for the study.

**S. No**.	**Oligo name**	**5' < —-Sequence—–>3'**	**Length**
1	GAPDH (F)	CCACCCATGGCAAATTCC	18
2	GAPDH (R)	TGGGATTTCCATTGATCACAAG	22
3	Dihydro folate reductase (F)	TGGTTCGCTAAACTGCATCGT	21
4	Dihydro folate reductase (R)	GGGCAGGTCCCCGTTCT	17
5	Cyclooxygenase-2 (F)	GAATCATTCACCAGGCAAATTG	22
6	Cyclooxygenase-2 (R)	TCTGTACTGCGGGTGGAACA	20
7	Lipoxygenase-5 (F)	GGGATGGACGCGCAAAG	17
8	Lipoxygenase-5 (R)	TTTACGTCGGTGTTGCTTGAGA	22
9	Hyaluronidase (F)	TGGGCCCCTATGTGATCAAT	20
10	Hyaluronidase (R)	ATGGCACCGCTGGTGACT	18
11	Ornithine decarboxylase (F)	CTGTCGTCTCAGTGTGAAATTCG	23
12	Ornithine decarboxylase (R)	CGCCCGTTCCAAAAGGA	17
13	Cathepsin D (F)	CACCACAAGTACAACAGCGACAA	23
14	Cathepsin D (R)	CGAGCCATAGTGGATGTCAAAC	22

### PI3K, AKT, mTOR, HDAC-6, VEGF, and HIF-1α Assays

The PI3K expression was determined using a kit procured from Bioassay Technology (Catalog No. E0896Hu). The protocol measured the AKT (Catalog No. 201-12-0893) and HDAC-6 (Catalog No. 201-12-2132) as indicated in the human-specific ELISA kit from Sun Red Company, China. mTOR (Catalog No. E-EL-H1655) was quantified by a kit purchased from ELab Sciences, USA. The VEGF (Catalog No. ab100663) and HIF-1α (Catalog No. ab171577) were estimated using a kit procured from Abcam, UK.

### Cell Cycle Analysis, Dichloro-Dihydro-Fluorescein Diacetate MMP, and Annexin V-FITC Estimation

The cell cycle analysis was carried out by the protocol reported by Fatima et al. (40). The cell lines were fixed with 70% ethanol. Before analysis, fixed cells were washed at least two times with PBS and PI solution containing RNase A to stain the cells. The samples were run on a flow cytometer within 1 h. Treated FaDu cells were incubated with 10 μM of DCFH-DA dye for 30 min then fluorescence was measured at 485/530 nm of emission/excitation. For FACS analysis, treated cells were incubated with 10 μM of DCFH-DA dye for 30 min, and the samples were analyzed within 1 h (41). The protocol for the analysis of MMP was similar to that of DCFH-DA except for Rhodamine 123 dye (41). The experiment was also carried out as per the method reported in Annexin-V-FITC apoptosis detection kit I (BD Pharmingen^TM^, Catalog No. 556547). For the assay, 1 × 10^6^ cells were grown in 6 well plates and treatment was given 24 h. The next day, cells were washed with cold PBS, and the pellet was resuspended in 100 μl (1X binding) buffer and left for 15 min followed by incubation with a dual fluorescent dye (5 μl FITC and 5 μl PI) for 10 min. The cell suspension was maintained upto 500 μl from the 1X binding buffer. The stained cells were analyzed by flow cytometer within 1 h (42).

### *In vivo* Anti-Tumor Activity Using S-180 Model and Toxicity Prediction

The anti-tumor activity of carvacrol was examined in mice S-180 (Sarcoma) model. The S-180 cells were collected from Swiss albino mice harboring 8–10 days old cells from the peritoneal cavity. On day 0, the S-180 cells (1 × 10^7^ cells/ml) were injected intra-peritoneal in non-inbred female Swiss albino mice (22–25 g, 8–10 weeks old) selected for the experiment. The next day (Day 1), the animals were randomized and divided into three treatment groups, one positive control group and one vehicle control group containing 5 animals in each group. The first three test groups were treated with 25, 50, and 75 mg/kg body weight (bw) i.p. dose of carvacrol for 1–9 days. The fourth group was given 5-fluorouracil; positive control at a dose of 20 mg/kg bw i.p. from day 1–9, and the fifth group (control) was administered normal saline (0.2 ml i.p.) for 9 days. On day 12, the animals were sacrificed, and the fluid was collected from each mouse's peritoneal cavity to evaluate tumor growth (43). The tumor growth inhibition was calculated using the following formula.


$$\begin{array}{l} \text{Tumor Growth Inhibition}\left(\%\right)=\frac{\left(\text{Number of tumor cells in control group}-\text{Number of tumor cells in the test group}\right)}{\text{Number of tumor cells in the control group}}\times 100 \end{array}$$

The physicochemical properties (ADMET) of carvacrol were calculated from SwissADME and Iazar toxicity prediction software.

### Statistical Analysis

The experimental results were calculated in MS-Excel, and the graphs were plotted on GraphPad version 5. The *in vitro* experiments were carried out in triplicate and represented as Mean ± SD (*n* = 3). Table curve 2D Windows version 4.07 was used to calculate IC_50_ values.

## Results

### Carvacrol Exerts Anti-Proliferative Activity Against Human Cancer Cell Lines

**Figure 1:**
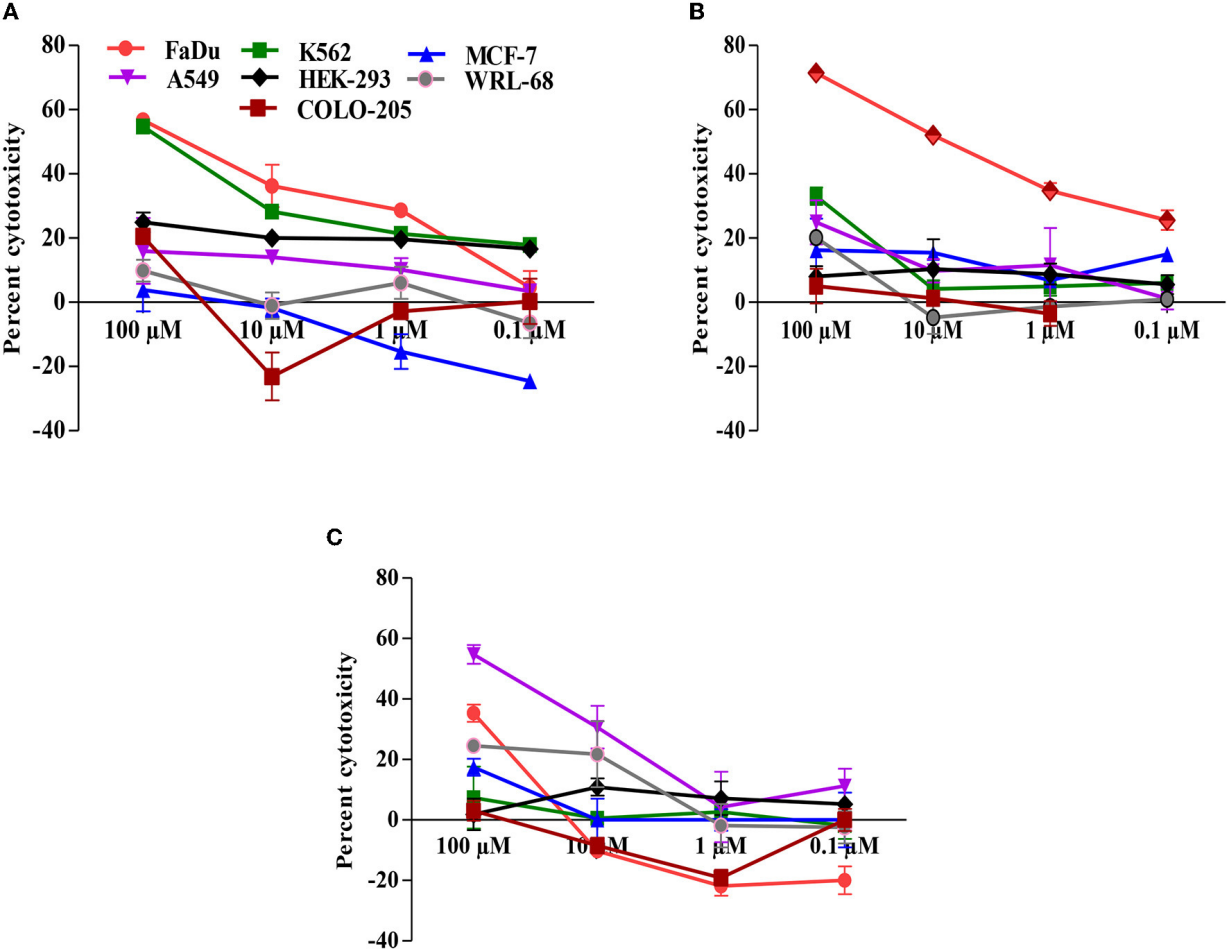
Carvacrol dose-dependently suppresses the growth of cancer cell lines. **(A)** Sulpho rhodamine B (SRB) dye was used to analyse the anti-proliferative effect of carvacrol in cancer cell lines. **(B)** 3-(4,5-dimethylthiazol-2-yl)-2,5-diphenyl tetrazolium bromide (MTT) dye was used to determine the effect of carvacrol in cancer cell lines. **(C)** Neutral red uptake (NRU) dye was used to detect the potential of carvacrol in cancer cell lines. Data are presented as mean ± SD (*n* = 3). Comparatively, carvacrol showed a potent effect against the FaDu cell line.

The anti-proliferative activity of carvacrol was investigated on a series of organ-specific human cancer/normal cell lines by employing SRB, MTT, and NRU assays (Figures 1A–C). In cell viability test, carvacrol exhibited IC_50_ value range from 9.61 ± 0.05 to 81.32 ± 11.83 μM in FaDu, K562, and A549 cell lines; however, the anti-proliferative effect was more pronounced in FaDu cell line with IC_50_ value 9.61 ± 0.05 and 42.01 ± 11.4 in MTT, and SRB assay, respectively (Table 2) and NRU assay, 35.32 ± 2.85% growth inhibition were observed. In addition, carvacrol reduced the proliferation of other cell lines (MCF-7, COLO-205, and WRL-68) up to 24% at the higher tested concentration (100 μM) and showed approximately 25% cell growth inhibition in HEK-293 (normal) cell line. These findings revealed that carvacrol affects the growth and proliferation of FaDu, K562, and A549 cell lines compared with other tested organ-specific cells. Therefore, further studies were performed with these cell lines to explore the mode of action of carvacrol.

**Table 2:** IC_50_ (μM) of carvacrol in cell-based assay.

	**FaDu**	**K562**	**A549**
SRB	42.01 ± 11.4	81.32 ± 11.83	–
MTT	9.61 ± 0.05	–	–
NRU	–	–	80.86 ± 8.28
DHFR	–	–	–
ODC	10.15 ± 0.04	12.66 ± 0.23	–
COX-2	21.86 ± 5.67	39.95 ± 10.24	–
LOX-5	82.56 ± 3.14	–	–
HYAL	88.11 ± 10.38	62.01 ± 8.4	–
CATD	–	–	–

*SRB, Sulphorhodamine B; MTT, 3-(4,5-dimethylthiazol-2-yl)-2,5-diphenyltetrazolium bromide; NRU, Neutral Red Uptake; DHFR, Dihydrofolate reductase; ODC, Ornithine decarboxylase; COX-2, Cyclooxygenase 2; LOX-5, Lipoxygenase 5; HYAL, Hyaluronidase; CATD, Cathepsin D; FaDU, Human squamous cell carcinoma epithelial cell line of hypopharynx; K562, Human myelogenous leukemia cell line; A549, Human adenocarcinoma alveolar basal epithelial cells*.

### Carvacrol Regulates Cells Proliferation by Suppressing ODC and HYAL Activities

**Figure 2:**
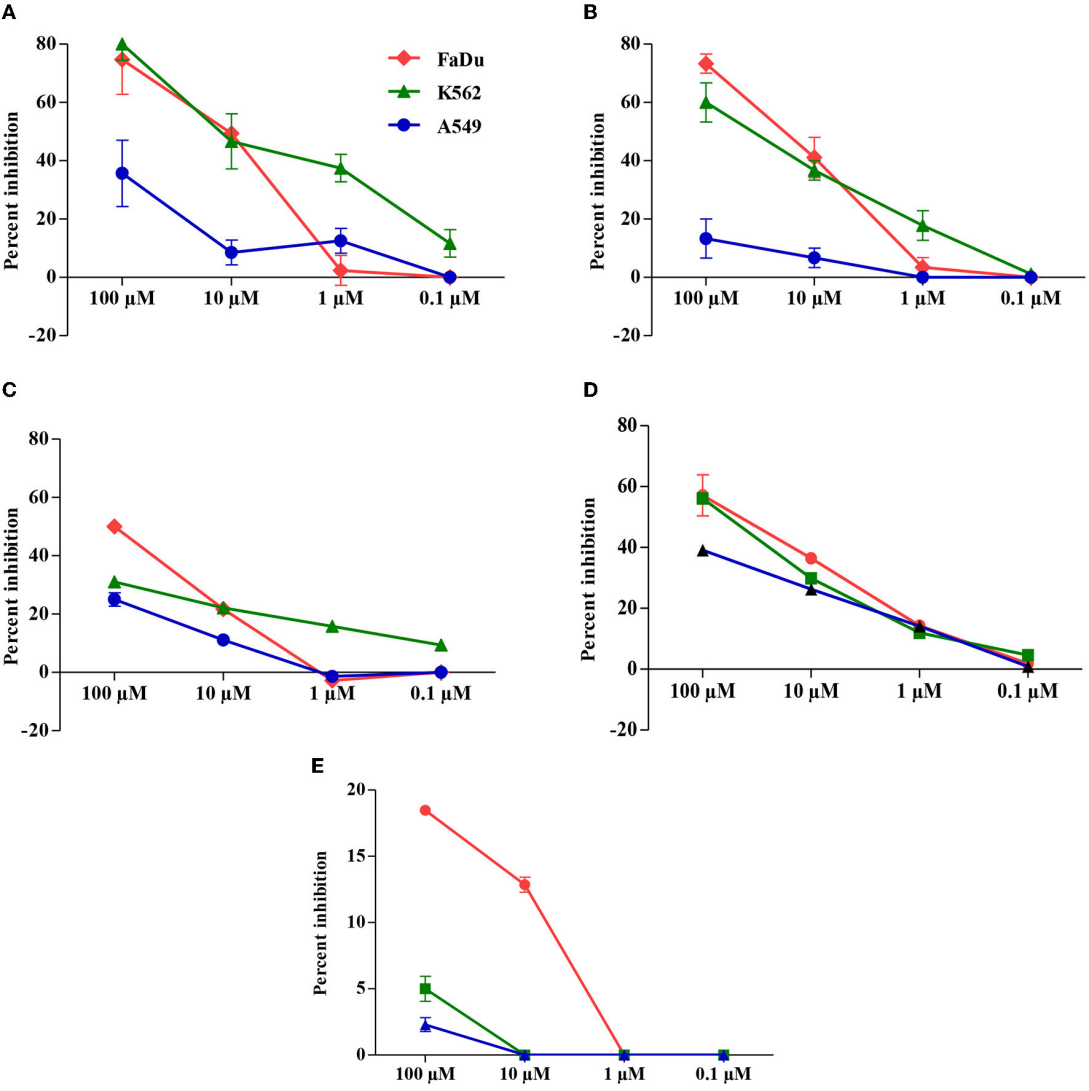
Carvacrol modulates the activity of cancer targets in a FaDu, K562, and A549 cell lines. Treated cancer cell lines were determined by the protocol described in materials and methods section. **(A)** Suppression of ornithine decarboxylase (ODC) activity by carvacrol. **(B)** Destruction of cyclooxygenase-2 (COX-2) activity by carvacrol. **(C)** Inflection of lipoxygenase 5 (LOX-5) activity by carvacrol. **(D)** Modulatory effect of carvacrol on hyaluronidase (HYAL) activity. **(E)** The suppression of cathepsin D (CATD) activity by carvacrol. The graph is shown in percentage inhibition. Data are presented as mean ± SD (*n* = 3). Comparatively, carvacrol exhibits a pronounced effect against ODC in FaDu cells.

**Figure 3:**
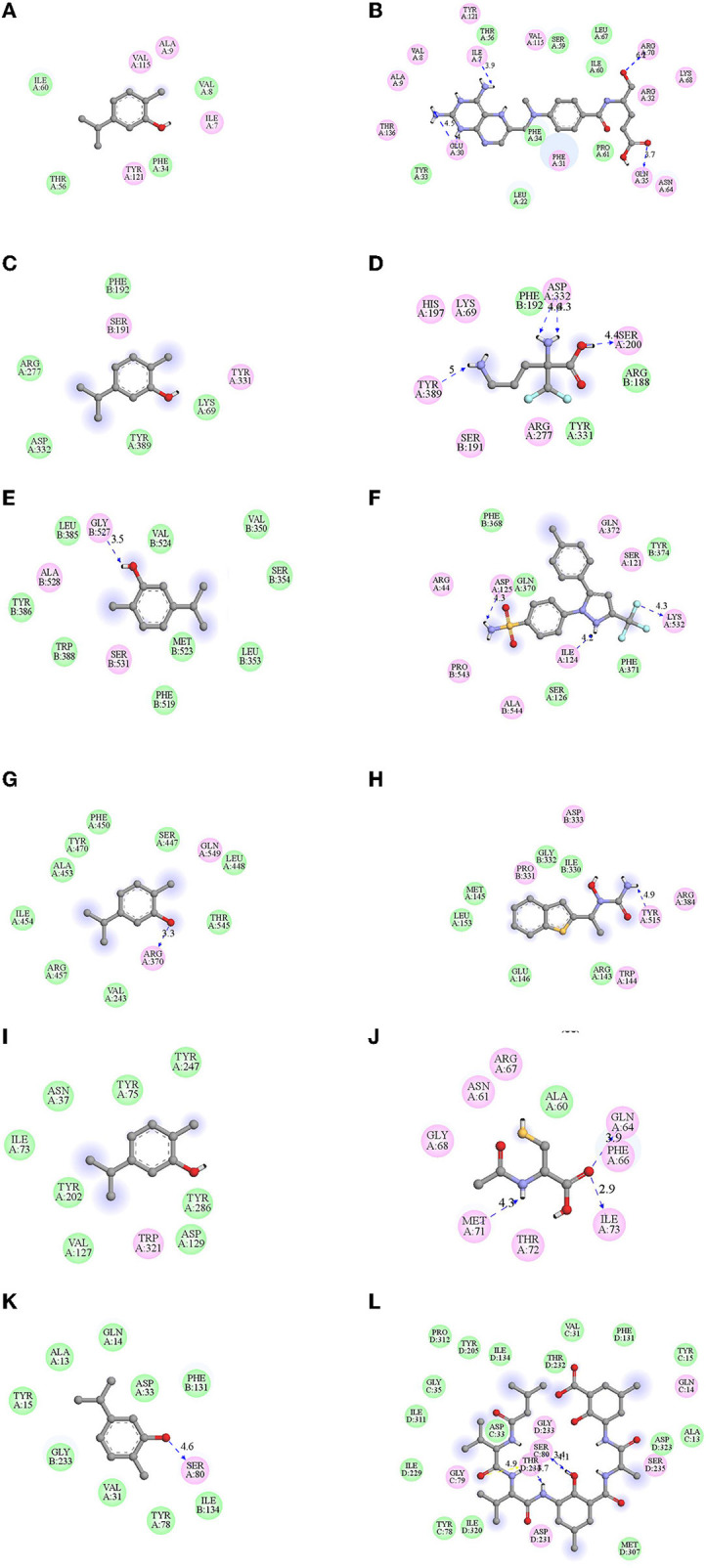
Docked pose of carvacrol with cancer targets were visualized by chimera and their hydrophobic interactions were observed by Discovery studio. Binding energies (BE) were obtained after the docking of carvacrol with the targeted receptor/proteins from AutoVina. Their interacting amino acid residues within 4Å of protein envisaged using discovery studio **(A)** dihydrofolatereductase (DHFR), **(B)** methotrexate (MTX), **(C)** ODC, **(D)** α-difluoro methyl ornithine (DFMO), **(E)** COX-2, **(F)** celecoxib, **(G)** LOX-5, **(H)** Zileuton, **(I)** HYAL, **(J)** N-acetyl cysteine (NAC), **(K)** CATD, **(L)** pepstatin A (PEP A). Carvacrol showed strong interaction with the selected targets, but the interaction of carvacrol with ODC is, even more, more potent than standard (DFMO).

The anti-proliferative activity of carvacrol was more pronounced in FaDu, K562, and A549 cell lines; thus, its effect was observed on different molecular targets responsible for cancer hallmarks in the cell-based test system. Carvacrol showed a non-significant effect on the overexpression of DHFR, it suppresses the enzyme action up to 7% at the higher tested concentration (100 μM). Although the molecular interaction of carvacrol with DHFR predicted a robust binding interaction with DHFR but not better than MTX, the binding pocket was also not similar to that of MTX (**Figures 3A,B**, Table 3). An inhibitory effect of carvacrol was observed with ODC activity with IC_50_ value of 10.15 ± 0.04 and 12.66 ± 0.23 μM in FaDu and K562 cell lines, respectively. In contrast, it reduces the ODC activity by 35.68% in A549 cell line at 100 μM (Figure 2A, Table 2). These results were validated by molecular docking studies wherein carvacrol possesses strong binding interaction with ODC compared with DFMO. However, the binding pocket was not similar (Figures 3C,D, Table 3). The effect of carvacrol was further investigated on COX-2 and LOX-5 activity. The results revealed that it retards the COX-2 activity with an IC_50_ value of 21.86 ± 5.67 and 39.95 ± 10.24 μM in FaDu and K562 cell lines, respectively. In contrast, it reduces COX-2 activity by 13.33% in the A549 cell line at 100 μM (Figure 2B, Table 2). The LOX-5 activity was above 50% in the FaDu cell line with an IC_50_ value of 82.56 ± 3.14 μM, and in K562 and A549 cell lines suppresses LOX-5 activity by <50% (Figure 2C, Table 2). Further, the molecular interaction of carvacrol with COX-2 and LOX-5 predicted a strong interaction with COX-2 but not robust than celecoxib. However, a strong interaction was observed with LOX-5 compared with zileuton. Although the binding pockets differed from standards, carvacrol acquires a strong interaction with COX-2 and LOX-5 (Figures 3E–H, Table 3). The effect of carvacrol was observed on HYAL and CATD activities. The results indicate that carvacrol inhibits HYAL activity with an IC_50_ value of 88.11 ± 10.38 and 62.01 ± 8.4 μM in FaDu and K562 cell lines, respectively, whereas it reduces the enzyme action by 39.15% in A549 cell line at 100 μM (Figure 2D, Table 2). The docking studies results suggest that the interaction of carvacrol with HYAL was stronger than NAC. Still, the binding pocket and interacting amino acids within 4Å of protein were not similar to NAC (Figures 3I,J, Table 3). Carvacrol suppressed CATD activity below 50%, i.e., 18.48 ± 0.33% in FaDu, 5 ± 0.94% in K562, and 2 ± 0.52% in A549 cell lines (Figure 2E). Additionally, the molecular docking interaction study revealed an interaction of carvacrol with CATD was not stronger than Pep A. Besides, the interacting amino residues were not similar in 4Å of the receptor (Figures 3K,L, Table 3).

**Table 3:** Molecular docking interaction of carvacrol with different cancer targets.

	**BE (Kcal/mol)**	**Ki (mM)**	**Residues within region of 4Å radius**	**H bonds forming residues bond length in Å**
DHFR	−6.6	1.44019E-05	VAL 115.A, ALA 9.A, VAL 8.A, ILE 7.A, PHE 34.A, TYR 121.A, THR 56.A, ILE 60.A	NO
MTX	−11.5	3.66366E-09	TYR 121.A, THR 56.A, ILE 7.A, VAL 115.A, SER 59.A, LEU 67.A, ILE 60.A, ARG 70.A, ARG 32.A, LYS 68.A, ASN 64.A, GLN 35.A, PRO 61.A, PHE 31.A, PHE 34.A, LEU 22.A, GLU 30.A, TYR 33.A, THR 136.A, ALA 9.A, VAL 8.A	3.9 Å with ILE 7.A; 3.7 Å with GLN 35.A; 4.54 Å with GLU 30.A; 6.2 Å with ARG 70.A
ODC	−5.8	5.56262E-05	ARG 277.A, SER 191.B, PHE 192.B, ASP 332.A, TYR 389.A, LYS 69.A, TYR 331.A	NO
DFMO	−5.3	0.000129439	HIS 197.A, LYS 69.A, PHE 192.B, ASP 332.A, SER 200.A, ARG 188.B, TYR 331.A, ARG 277.A, SER 191.B, TYR 389.A	4.6 Å, 4.3 Å with ASP 332.A;4.4 Å with SER 200.A; 5 Å with TYR 389.A
COX-2	−6.6	1.44019E-05	VAL 524.B, VAL 350.B, SER 354.B, LEU 353.B, PHE 519.B, MET 523.B, SER 531.B, TRP 388.B, TYR 386.B, ALA 528.B, LEU 385.B, GLY 527.B	3.5 Å with GLY 527.B
Celecoxib	−8.7	4.14876E-07	PHE 368.B, GLN 372.A, SER 121.A, TYR 374.B, LYS 532.A, PHE 371.A, ILE 124.A SER 126.A, ALA 544.B, PRO 543.B, ARG 44.A, ASP 125.A, GLN 370.A	4.3 Å with LYS 532.A;4.2 Å with ILE 124.A; 4.3 Å with ASP 125.A
LOX-5	−7.0	7.32811E-06	ALA 453.A, TYR 470.A, PHE 450.A, SER 447.A, GLN 549.A, LEU 448.A, THR 545.A, ARG 370.A, VAL 243.A, ARG 457.A, ILE 454.A	3.3 Å with ARG370.A
Zileuton	−6.5	1.70521E-05	PRO 331.B, GLY 332.B, ILE 330.B, ASP 333.B, TYR 515.A, ARG 384.A, ARG 143.A, TRP 144.B, GLU 146.A, LEU 153.A, MET 145.A	4.9 Å with TYR 515.A
HYAL	−6.5	1.70521E-05	ILE 73.A, ASN 37.A, TYR 75.A, TYR 247.A, TYR 286.A, ASP 129.A, TREP 321.A, VAL 127.A, TYR 202.A	2.72 Å with ASN 37.A
NAC	−4.8	0.000301198	GLY 68.A, ASN 61.A, ARG 67.A, ALA 60.A, GLN 64.A, PHE 66.A, ILE 73.A, THR 72.A, MET 71.A	3.9 Å with GLN 64.A; 2.9 Å with ILE 73.A; 4.3 Å with MET 71.A
CATD	−5.8	5.56262E-05	TYR 15.A, ALA 13.A, GLN 14.A, ASP 33.A, PHE 131.B, SER 80.A, ILE 134.B, TYR 78.A, VAL 31.A, GLY 233.B	4.6 Å with SER 80.A
Pep A	−8.9	2.9594E-07	PRO 312.D, TYR 205.D, ILE 134.D, THR 232.D, VAL 31.C, PHE 131.D, TYR 15.C, GLN 14.C, ALA 13.C, ASP 323.D, SER 235.D, MET 307.D, ASP 231.A, THR 233.D, SER 80.C, GLY 233.D, ASP 33.C, GLY 79.C, ILE 320.D, TYR 78.C, ILE 229.D, ILE 311.D, GLY 35.C	3.4 Å, 4.1 Å with SER 80.C; 4.7 Å, 4.9 Å with THR 233.D

*DHFR, Dihydro folate reductase; MTX, Methotrexate; ODC, Ornithine decarboxylase; DFMO, α-difluoro methyl ornithine; COX-2, Cyclooxygenase 2; LOX-5, Lipoxygenase 5; HYAL, Hyaluronidase; NAC, N-acetyl cysteine; CATD, Cathepsin D; Pep A, Pepstatin A; BE, Binding energy; Ki, Inhibition constant*.

**Figure 4:**
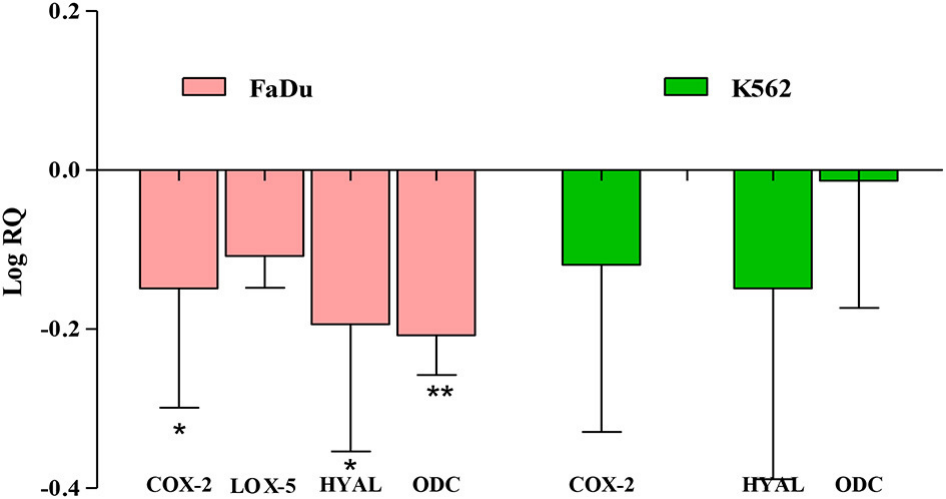
Carvacrol affects the transcriptional level of *HYAL* and *ODC* in FaDu cell line. The mRNA expression of the tested gene in treated and non-treated FaDu and K562 cell lines were analyzed using real-time qPCR. Glyceraldehyde 3-phosphate dehydrogenase (GAPDH) was used as an internal control. Data are presented as mean ± SD (*n* = 3). Carvacrol potently downregulates the expression of ODC in the FaDu cell line (*p < 0.05, **p < 0.01).

These results suggest that carvacrol suppresses COX-2, LOX-5, HYAL, and ODC activities in FaDu cell line and COX-2, ODC, and HYAL in K562 cell line by more than 50%. The molecular interaction studies revealed that carvacrol showed good binding with the selected targets; thus, its efficacy was further validated by the real-time expression analysis. The data indicate that carvacrol downregulates the expression of COX-2 in both the tested cell lines but the fold change in comparison to control was more in the FaDu cell line (1.40) followed by the K562 cell line (1.31). In addition, carvacrol diminishes the expression of LOX-5 in the FaDu cell line with a fold change of 1.28. Similarly, carvacrol downregulates the expression of HYAL and ODC in FaDu cell line with a fold change of 1.56 and 1.61, respectively. However, in K562 cells, carvacrol downregulates the expression by 1.03 and 1.40 for ODC and HYAL, respectively (Figure 4). Comparatively, carvacrol was found most effective against FaDu cells; thus, further mode of action was investigated in FaDu cells.

### Carvacrol Exhibits a Non-Significant Change in PI3K, AKT, mTOR, HDAC-6, VEGF, and HIF-1α Activities

**Figure 5:**
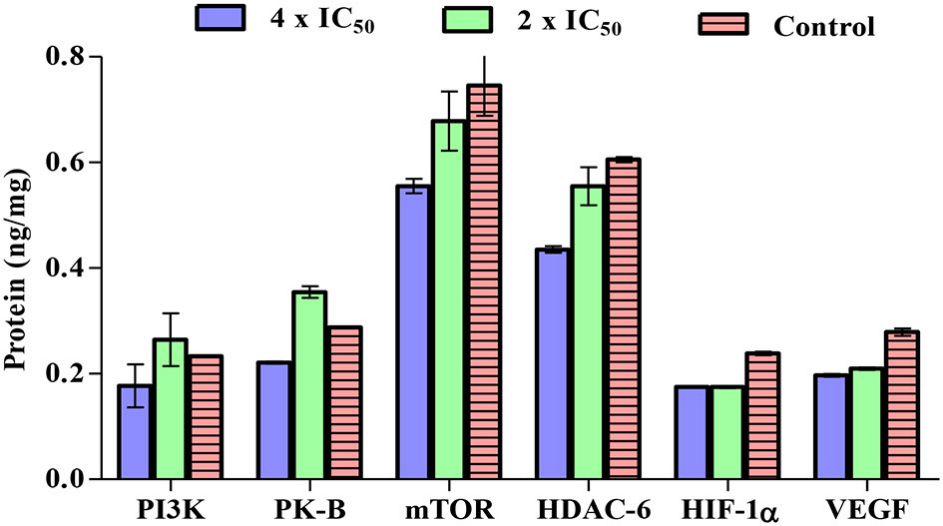
Carvacrol effects were examined on phosphotidyl inositol-3 kinase (PI3K)/protein kinase B (AKT)/mammalian target of rapamycin (mTOR), histone deacetylase (HDAC)-6, vascular endothelial growth factor (VEGF)/HIF-1α in FaDu cell line. FaDu cells were treated with carvacrol, radioimmuno precipitation assay (RIPA) buffer was used to extract crude protein, and the ELISA kits were used to analyse its inhibitory activity. The effect of carvacrol was non-significant on these targets.

The potential of carvacrol was examined against these markers in FaDu cell line. Carvacrol suppresses the activity of the PI3K/AKT at the higher tested concentration; however, at lower concentrations, the inhibition and activation of the enzyme were non-significant. In mTOR, carvacrol reduces the activity and inhibits HDAC-6 activity in a dose-dependent manner. Carvacrol suppresses the VEGF and HIF-1α activity at both the tested concentrations, but the effect was non-significant (Figure 5).

### Carvacrol Arrest G2/M Phase in FaDu Cells Deteriorates Membrane Potential and Non-Significantly Increases the ROS Production

**Figure 6:**
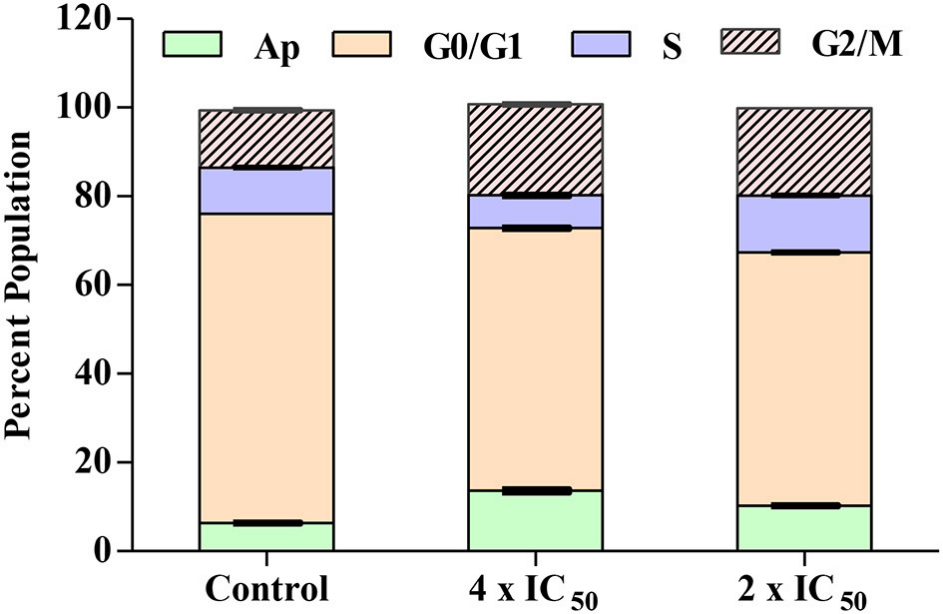
Carvacrol arrest the G2/M phase of FaDu cells. Treated FaDu cells were stained with propidium iodide (PI) to determine the arrest of the diverse phase of cells by following a protocol written in the materials and methods section. FACS Diva software was employed to set parameters (SSC, FSC, and PI) and gated concerning untreated FaDu cells. Carvacrol arrests the G2/M phase and increases the number of sub-diploid populations.

**Figure 7:**
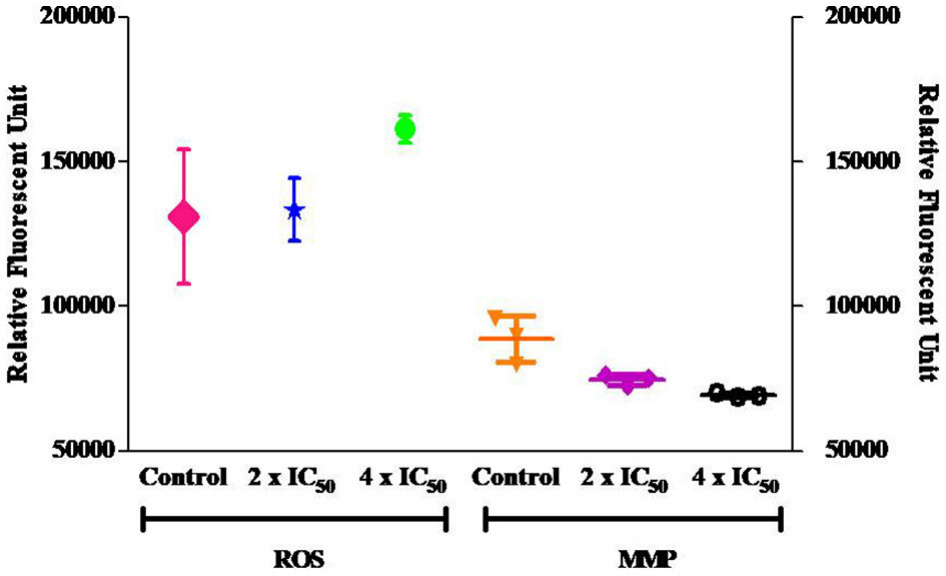
Carvacrol non-significantly increase the reactive oxygen species (ROS) production and decreases the mitochondrial membrane potential (MMP) in FaDu cells. One set of treated FaDu cells was stained with DCFDA dye, and another set was stained with rhodamine 123 dye, followed by analysis *via* fluorimeter.

**Figure 8:**
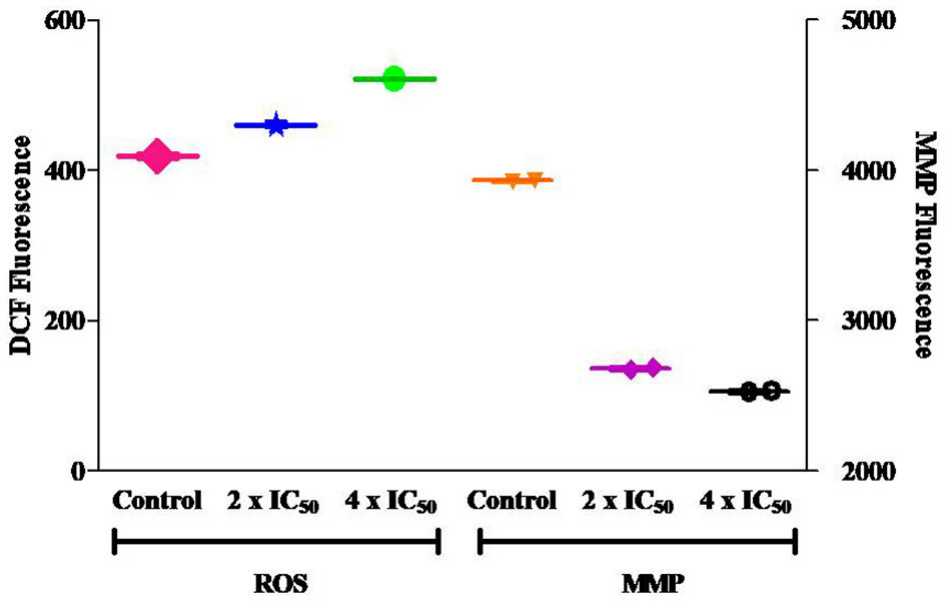
Carvacrol non-significantly affect ROS and MMP in FaDu cells. One set of treated FaDu cells was stained with DCFDA dye, and another set was stained with rhodamine 123 dye, followed by analysis by flow cytometer.

Carvacrol exhibits potent anti-proliferative activity against the FaDu cell line in *in vitro* tests. Cell cycle analysis was conducted to explore its effect on diverse cell cycle phases and the number of sub-diploid populations. The results confirmed that carvacrol arrests the G2/M phase of FaDu cells in a dose-dependent manner and raises the sub-diploid population number at the tested concentrations (Figure 6). The efficacy of carvacrol was observed in inducing ROS production and disrupting MMP in FaDu cell line. The results suggest that carvacrol increases the ROS production in FaDu cell line in a dose-dependent manner; however, the effect was non-significant (Figure 7). Moreover, the flow cytometer detected a similar result wherein carvacrol increases the intracellular ROS in FaDu cell line (Figure 8). In the MMP assay, carvacrol diminishes the membrane potential of the FaDu cell line at the tested concentrations (Figure 7). Carvacrol treated FaDu cell population shifted from red to green channel, indicating a dose-dependent decline in the potential of mitochondrial membrane; however, the effect was non-significant (Figure 8).

### Carvacrol Causes Late Apoptosis and Necrosis in FaDu Cells

**Figure 9:**
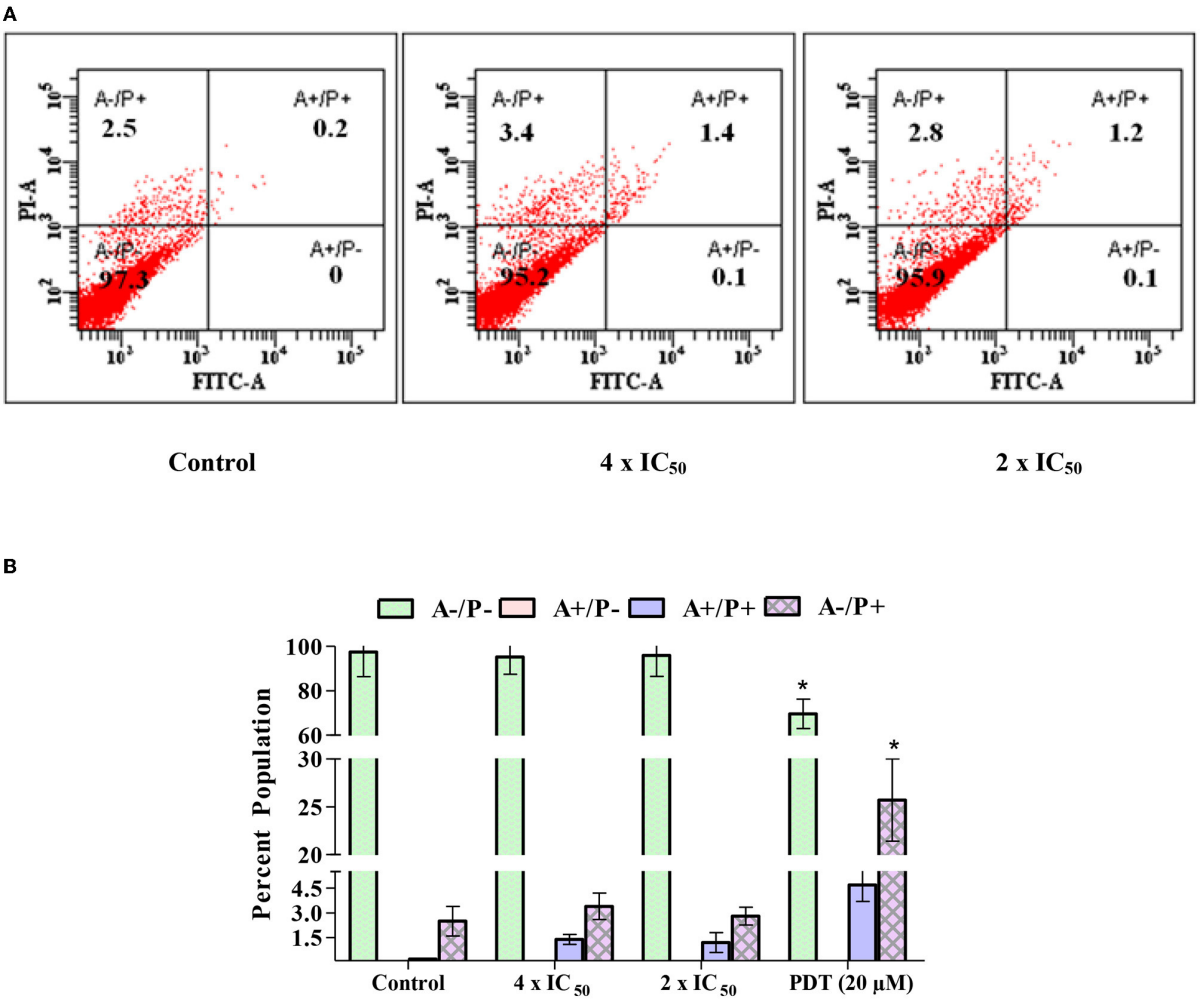
**(A,B)** Carvacrol induces late apoptosis and necrosis of FaDu cells. FaDu cells were treated with carvacrol and stained with Annexin V-fluorescein isothiocyanate (FITC) and PI to determine apoptosis and necrosis through flow cytometer. The effect of carvacrol was non-significant and PDT was significant (*p < 0.05).

The FaDu cells treated with carvacrol and non-treated cells were used to determine whether it exhibits apoptosis and necrosis or its effect is limited to the quiescent stage. At higher concentrations, carvacrol promotes early apoptosis (0.1%), late apoptosis (1.4%), and necrosis (3.4%); however, in non-treated cells, no early apoptosis was observed, the percentage of late apoptosis and necrosis were 0.2 and 2.5%, respectively (Figures 9A,B).

### Carvacrol Inhibits Tumor Growth of Mice in Sarcoma-180 (S-180) Model

Sarcoma-180 is an extensively used model in tumor biology to find the efficacy of anti-cancer agents. Therefore, the potency of carvacrol was analyzed in sarcoma cells; 1 × 10^7^ sarcoma cells were transplanted into the peritoneal cavity of mice, 75, 50, and 25 mg/kg bw doses of carvacrol were administered. Interestingly, no adverse effect on mice bw was noticed, and carvacrol showed significant prevention of tumor growth by 72.62 and 55.94% at 75 and 50 mg/kg bw *i.p*. dose, respectively. In contrast, at 25 mg/kg bw, only 28.12% tumor reduction was observed. However, 5-fluorouracil exhibited 93.75% tumor growth reduction at 20 mg/kg bw. During the experimental period, no mortality was noticed, and the percentage inhibition of the tumor was calculated by comparing control values of S-180 tumor cells (Table 4).

**Table 4:** *In vivo* activity of carvacrol against Sarcoma-180 model.

**Test sample**	**Dose (mg/kg bw i.p.)**	**Tumor volume (mL) Mean ± S.E**	**Tumor weight (g) Mean ± S.E**	**Tumor cell count (1 × 10^**7**^) Mean ± S.E**	**Tumor growth inhibition (%)**
Carvacrol	25	3.5 ± 0.54	3.36 ± 0.60	57.5 ± 8.72	28.12
	50	1.57 ± 0.25^**^	1.39 ± 0.33^*^	35.25 ± 4.68^**^	55.94
	75	1.16 ± 0.34^**^	1.10 ± 0.41^**^	21.9 ± 1.78^**^	72.62
5 FU	20	0.16 ± 0.01^***^	0.16 ± 0.01^***^	5 ± 0.28^***^	93.75
Control	NS (0.2 mL)	4.08 ± 0.52	3.91 ± 0.56	80.05 ± 7.92	—

* *Significant (p < 0.05)*,

** *Highly significant (p < 0.01)*,

*** *Very highly significant (p < 0.001). Data are mean ± S.E (n = 5)*.

### *In silico* Toxicity Assessment by SwissADME and Iazar

Carvacrol was further evaluated for its physicochemical properties (ADME) by SwissADME and Iazar prediction software. The prediction studies indicate that carvacrol has a molecular weight of <500, the number of hydrogen bond donors, acceptor, and values of Log *p* was <5. The topological polar surface area (TPSA) and rotatable bond were also within the acceptable range. The value of log *p* is responsible for transportation, implying hydrophobicity, and lipophilicity (44). The analysis showed that carvacrol has adequate hydrophobic and moderate lipophilic properties, quickly crossing the membrane surface. Due to its moderate lipophilicity and TPSA, it can quickly arrive at the receptor site. The solubility (Log S) of carvacrol considerably affects its absorption and distribution features (45).

Interestingly, the Log S value of the carvacrol was also within the acceptable range, and gastrointestinal absorption of carvacrol was also high. Carvacrol was also predicted as non-mutagenic on *Salmonella typhimurium* and exhibited non-carcinogenic activity. The *in silico* toxicity assessment results suggest that carvacrol follow the Lipinski rule of five; therefore, it can be considered as a safe molecule (Table 5).

**Table 5:** *In silico* prediction studies of carvacrol from SwissADME and Iazar.

**Chem I.D**.	**10,364**
Molecular formula	C_10_H_14_O
Molecular weight	150.22
X Log P3	3.49
Log S	−3.31
Hydrogen bond donor	1
Hydrogen bond acceptor	1
Rotatable bond count	1
Topological polar surface area	20.23 A^2^
GI absorption	High
Log *K*_p_ (skin permeation)	−4.74 cm/s
Lipinski rule	Yes; 0 violation
Carcinogenicity (Rat)	None
Mutagenicity (*Salmonella typhimurium*)	Non-mutagenic

## Discussion

Cancer hallmarks acquired evolutionary-advantageous characteristics that complementarily promote the transformation of phenotypically normal cells into malignant ones and promote the progression of malignant cells while exploiting host tissue (46). Hanahan and Weinberg described six hallmarks in 2000, and later on, in 2011, four more hallmarks were added to the list (47, 48). However, most anti-cancer drugs target hallmark, but few are effective for a limited duration (46). Thus, there is a need to identify a molecule that targets cancer hallmarks for a prolonged period and inhibits cell growth. The results of Figures 1A–C revealed that carvacrol suppresses the growth of FaDu, K562, and A549 cell lines which are in agreement with the published report on murine B16 melanomas (13), human A549 non-small lung cancer (15), Hep-2 human larynx carcinoma cells (49), gastric carcinoma cells (50), leiomyosarcoma cells (17), MDA-MB-231 human metastatic breast cancer cells, MCF-7 chemosensitive breast cancer cell line, hepatocellular carcinoma (51), and human erythroleukemic K562 cell line (18). Dai et al. reported the anti-tumor activity of carvacrol in different cell lines (52), and Subramaniyan et al. showed that carvacrol possesses the potent anti-proliferative activity by obstructing the cell growth and averting metastasis in diethyl nitrosamine-induced hepatocellular carcinogenesis (53). However, so far, no report is accessible with FaDu cell line, and limited pieces of evidence are available on K562 and A549 cell lines; hence, we systematically investigated the anti-proliferative potential of carvacrol with the selected targets of cancer hallmarks.

In a normal cell, irreparable DNA damage and uncontrolled proliferation involve the activation of apoptotic machinery results in cell death, but in the cancer cells, the balance between cell proliferation and cell death that usually maintains a healthy tissue homeostasis is disturbed (54). The disturbance may be due to the overexpression or hypersecretion of the enzymes/proteins and altered signaling pathway. The modulation of enhanced enzyme activity and protein level can help diminish cancer hallmarks, such as proliferation, angiogenesis, metastasis, inflammation, invasion, and reduced apoptosis. ODC is a chief controller of cellular proliferation, and decreased apoptosis catalyzes ornithine conversion to polyamines. These polyamines are essential for modulators of ion transport channels, nucleic acid synthesis, DNA double-strand break repair pathway, and protein synthesis regulation (55). DHFR reduces dihydrofolic acid to tetrahydrofolic acid and helps in the biosynthesis of DNA bases responsible for cell proliferation (56). COX-2 and LOX-5 accumulate at the inflammation sites produced in AA metabolism, an intermediate product of diacylglycerol (40). HYAL is a glycosylated protein cleaves at β-D 1–4 linkage of hyaluronan (HA) in the extracellular matrix. The fragments of HA increase the elasticity and adhesion of the cells. Thus, an overexpression of HYAL is liable for invasion, angiogenesis, inflammation, and metastasis (57). CATD is an aspartic lysosomal endopeptidase that degrades proteins. Usually, it becomes active in lysosomes wherein pH is 3–3.5, but in cancer cells, it becomes active in the cytosol (58). The previous literature suggest unusual expression of selected targets enhanced the proliferation, invasion, angiogenesis, metastasis, and migration of the cancer cells at varied sites; therefore, these are considered significant cancer hallmarks (40).

Thus, the efficacy of carvacrol was observed on different targets and the analysis of results indicate that carvacrol more potently inhibits the proliferation of FaDu cells by downregulating the expression of ODC and HYAL. ODC activity is determined by the method, which is based on the enzyme that transforms L-ODC (substrate) to yellow colored putrescine (product) soluble in 1-pentanol, the absorbance of which was measured spectrophotometrically (32). In addition, carvacrol halts the growth of K562 cells by diminishing the expression of HYAL, angiogenesis marker; however, it was not effective in transforming the tested targets in A549 cell line. It indicates that the growth inhibition of A549 cell line may be due to off-target or pleiotropic action in the intermediate steps of signaling pathway/metabolites. Carvacrol found in the essential oil of *Origanum vulgare* has been reported to be endowed with anti-skin aging activity by inhibiting HYAL, collagenase, and elastase expression (59). However, carvacrol downregulates the expression of Bcl-2 gene and upregulate the expression of Bax gene in MCF-7 cells (60).

Any deregulation of the cell cycle and checkpoint disruption is crucial; therefore, a halt in any phase of the cell help in stopping the division. Thus, the cell cycle analysis was carried out, followed by ROS and MMP measurement. An enhanced ROS level and decreased MMP releases cytochrome c in the cytoplasm, wherein it binds with apaf protein and pro-caspase-9 to form apoptosome. This formation activates caspase-9, which in turn activates caspase-3, due to which cells undergo apoptosis. It was revealed that carvacrol arrest the G2/M phase of FaDu cells, increases the sub-diploid population and non-significantly affect the basal level of ROS and MMP. This observation is contrary to the earlier published report on the carvacrol-induced apoptosis of MDA-MB-231 cells by decreasing MMP, which activates caspase by cleaving poly-ADP-ribose polymerase [PARP; (19)]. Apoptosis represents one of the significant hallmarks for most or all types of cancer. During the onset of apoptosis, the phosphatidylserine (PS) lying on the inner surface of the plasma membrane moves to the outer surface, which has a robust binding affinity for Annexin V while fluorescein isothiocyanate (FITC) bound to Annexin V and a fluorescence green color indicate apoptosis of the cells. PI is a fluorescent intercalating agent used to label the cellular DNA in a necrotic cell (38). The cell cycle data support that carvacrol induces late apoptosis and necrosis of FaDu cells. Overall, experimental findings revealed that the suppression of FaDu cell proliferation could be due to the modulation of ODC and HYAL activities.

Head and neck cancer squamous cell carcinoma (HNSCC) is the sixth most common type of cancer globally. Approximately 550,000 morbidity and 300,000 mortality due to HNSCC are reported each year, and the survival rate reported in 5 years varies from 40 to 50% (61). The major drawback of HNSCC is its identification at later stages of metastasis/invasion, and the available treatments include paclitaxel, cisplatin, etc., but the survival rate is significantly less (62). Thus, the inhibition of FaDu cell proliferation by carvacrol is revealed through the downregulation of ODC and HYAL expressions, potential cancer hallmarks for proliferation, metastasis, and invasion.

Besides *in vitro* experiments, carvacrol ability was also observed *in vivo* in anti-tumor model. Sarcoma-180 is one of the most extensively used models in tumor biology to discover and develop anti-cancer agents (43). Carvacrol retards the tumor growth of S-180 cells by more than 70% at higher tested dose. Toxicity is the foremost drawback of anti-cancer agents/drugs; hence, the Ghose and Lipinski rules were developed to identify potent anti-cancer agents with lesser toxicity. The carvacrol's physicochemical property (ADMET) exhibits drug-likeness property and follows Lipinski's rule of 5, suggesting it could be considered as a safe molecule. An earlier report also advocates the non-toxic nature of carvacrol and its use in reducing the adverse effects of chemotherapy. Irinotecan hydrochloride, an anticancer drug, trigger inflammation, induces intestinal mucositis and causes cell damage through the TRPC channel, subfamily A, member 1 receptor (51). Carvacrol acts as an agonist of the TRPC channel and reduces inflammation by diminishing the activity of NFκB and COX-2 in mice (63).

## Conclusion

Carvacrol possesses significant anti-proliferative activity against FaDu, K562, and A549 cell lines. The molecular and cell-target based assays and expression analysis revealed that carvacrol inhibits HYAL and ODC activities in FaDu cell line, due to which the cells may undergo apoptosis. Besides *in vitro* system, carvacrol was found effective in reducing the growth of mice (S-180) tumor *in vivo*. The *in silico* prediction also indicates that carvacrol can be considered a safe molecule. Thus, the findings advocate that carvacrol could be used as an adjuvant for inhibiting cancer hallmarks, proliferation, metastasis, invasion, and angiogenesis in hypopharyngeal carcinoma cells. Further, it can be exploited as a pharmacophore for developing a safe and effective multi-targeted anti-cancer medicament.

## Data Availability Statement

The original contributions presented in the study are included in the article/Supplementary Material, further inquiries can be directed to the first/corresponding author/s.

## Ethics Statement

The animal study was reviewed and approved by Institutional Animal Ethics Committees (IAEC) of the CSIR-Central Institute of Medicinal and Aromatic Plants, Lucknow Uttar Pradesh, India (Approval No. CIMAP/IAEC/2019-2021/04).

## Author Contributions

KF: formal analysis specifically conducting *in vitro* and *in vivo* experiments and data collection. AM: validation and *in silico* analysis and draft manuscript writing. SL: idea, planning, data curation, and final manuscript. All authors contributed to the article and approved the submitted version.

## Funding

The authors acknowledge The Council of Scientific and Industrial Research-Aroma Mission (Catalyzing rural empowerment through cultivation, processing, value addition, and marketing of aromatic plants, HCP 007) and Science and Engineering Research Board, New Delhi (EEQ/2021/000292) for supporting this work. KF acknowledges the Council of Scientific and Industrial Research (CSIR), New Delhi, for her Senior Research Fellowship.

## Conflict of Interest

The authors declare that the research was conducted in the absence of any commercial or financial relationships that could be construed as a potential conflict of interest.

## Publisher's Note

All claims expressed in this article are solely those of the authors and do not necessarily represent those of their affiliated organizations, or those of the publisher, the editors and the reviewers. Any product that may be evaluated in this article, or claim that may be made by its manufacturer, is not guaranteed or endorsed by the publisher.

## Supplementary Material

Supplementary Figure 1 Anti-proliferative effect of carvacrol on hypopharyngeal carcinoma cells.

## Abbreviations

AA: arachidonic acid
Ab/Am: antibiotic/antimycotic
BE: binding energy
BSA: bovine serum albumin
CATD: cathepsin D
COX-2: cyclooxygenase-2
DFMO: α-difluoro methyl ornithine
DOXO: doxorubicin
CM-H2DCFDA: chloromethyl derivative of dichlorofluorescindiacetate
DMEM: Dulbecco's minimal essential media
DHFR: dihydrofolatereductase
DCFDA, 2′: 7′ dichlorofluorescindiacetate
DNA: deoxyribonucleic acid
DMSO: dimethyl sulphoxide
ELISA: enzyme-linkedimmunosorbent assay
EDTA: ethylenediamine tetra acetic acid
FBS: fetal bovine serum
FACS: fluorescence-activated cell sorting
GAPDH: glyceraldehyde 3-phosphate dehydrogenase
HEPES: N-2-hydroxy ethyl piperazine-N′-2-ethanesulfonic acid
HYAL: hyaluronidase
HA: hyaluronic acid
HDAC: histone deacetylase
IC_50_: half maximal inhibitory concentration
IDT: Integrated DNA Technologies
Ki: inhibitory constant
LOX-5: lipoxygenase 5
LDH: lactate dehydrogenase
MTT: 3-(4, 5-dimethylthiazol-2-yl)-2,5-diphenyl tetrazolium bromide
MTX: methotrexate
mTOR: mammalian target of rapamycin
NAC: N-acetyl cysteine
MMP: mitochondrial membrane potential
NADPH: nicotinamide adenine dinucleotide phosphate hydrogen
NRU: neutral red uptake
NaOH: sodium hydroxide
ODC: ornithine decarboxylase
PEP A: pepstatin A
PI3K: phosphotidyl inositol-3 kinase
PKB/AKT: protein kinase B
PI: propidium iodide
PDB: protein data bank
PCR: polymerase chain reaction
PBS: phosphate buffer saline
RPMI: roswell park memorial Institute
ROS: reactive oxygen species
RNA: ribonucleic acid
RNase A: ribonuclease A
Rh123: rhodamine 123
RIPA: radioimmuno precipitation assay
RBC: red blood cells
SRB: sulphorhodamine B
TPA: 12-O-tetradecanoyl phorbol-13-acetate
TCA: tricarboxylic acid
TMPD, N, N, N′: N′-tetra methyl-p-phenylenediamine
TNBS: tri nitro benzene sulphonic acid.
