# Supplementary figures and images for: Carvacrol Arrests the Proliferation of Hypopharyngeal Carcinoma Cells by Suppressing Ornithine Decarboxylase and Hyaluronidase Activities
Source: Front Nutr. 2022 Apr 8;9:857256. doi: 10.3389/fnut.2022.857256 (PMC9028219; doi:10.3389/fnut.2022.857256)

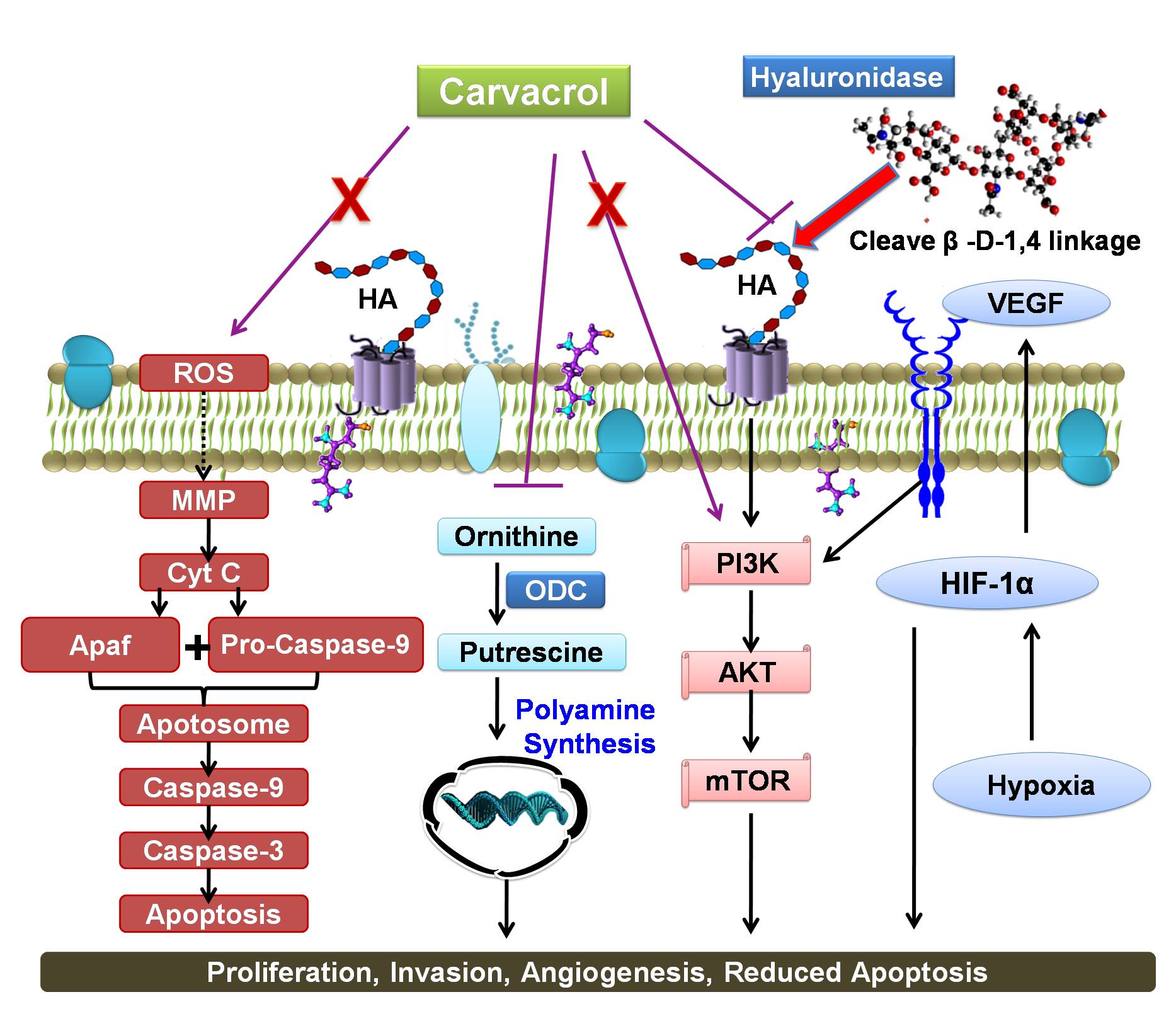

Supplement: Supplementary Figure 1 — Anti-proliferative effect of carvacrol on hypopharyngeal carcinoma cells. [file Image_1.JPEG]
